# Effects of Ipilimumab on expanded tumor infiltrating lymphocytes in patients with stage IV malignant melanoma

**DOI:** 10.1186/2051-1426-1-S1-P241

**Published:** 2013-11-07

**Authors:** Jon Bjørn, Marco V Donia, Rikke Andersen, Sine R Hadrup, Inge Marie Svane

**Affiliations:** 1 Center for Cancer Immune Therapy, Herlev University Hospital, Herlev, Denmark; 2 Department of Oncology, Herlev University Hospital, Herlev, Denmark

## Introduction

Adoptive cell therapy, where patients are treated with expanded autologous T cells derived from tumor specimens, is currently one of the most effective treatments for stage IV malignant melanoma. Clinical studies have indicated a link between treatment with anti-CTLA-4 treatment (e.g. Ipilimumab) prior to tumor removal, and a favorable treatment response to subsequent adoptive cell therapy.

## Methods

In the current study we compared immunological phenotype and functionality of T cells, generated from melanoma biopsies, from Ipilimumab naïve patients with patients that had received treatment with Ipilimumab within six month prior to tumor removal. Tumor biopsies were obtained prospectively from 32 consecutive patients (16 treated with Ipilimumab within six months from tumor removal and 16 Ipilimumab naive), and T cells were cultured and expanded according to the rapid expansion protocol. Expanded cells were stained for intra- and extracellular markers and subjected to flow cytometric analysis. Additionally, combinatorial coding with MHC-I multimers and co-culture assays with autologous tumor cells were performed in order to assess tumor-specific responses.

## Results

Preliminary results showed that prior Ipilimumab treatment did not affect the duration of time to establish young TIL cultures or expansion capacity of cultured T cells. Analysis for several phenotypic markers did not reveal any significant differences related to prior treatment, though a trend towards higher frequency of CD8+CD28+ T cells was demonstrated in patients treated with Ipilimumab. Additionally, there was no difference in frequency of tumor reactive T cells in expanded cultures. Combinatorial coding with MHC-I multimers revealed frequent responses toward common tumor associated antigens including MART-1, gp100, TRP-2, Tyrosinase and MAGE. Experiments are ongoing and assessment of possible treatment-related differences, in terms of antigen specificity or tumor reactivity will be presented.

## Conclusions

Despite indications of a favorable effect of Ipilimumab treatment prior to adoptive cell therapy, our preliminary results indicate that expanded TILs from ipilimumab treated and untreated patients possess comparable quantitative, phenotypic and functional characteristics. Additional experiments are needed to further scrutinize changes in TILs as well as tumor and microenvironment which could lead to increased clinical response to adoptive T cell therapy in ipilimumab treated patients.

